# LIMA-LAD Steal Syndrome as a Cause of Post-CABG Angina

**DOI:** 10.1016/j.jaccas.2025.104828

**Published:** 2025-08-27

**Authors:** Naveen S. Multani, Ziad R. Affas, Patrick B. Alexander

**Affiliations:** a Internal Medicine Department, Henry Ford Providence Hospital, Southfield, Michigan, USA; b Cardiology Department, Henry Ford Providence Hospital, Southfield, Michigan, USA

**Keywords:** chest pain, coronary artery bypass, myocardial ischemia, nuclear medicine, occlusion, percutaneous intervention

## Abstract

**Background:**

Coronary steal from unligated side branches of the left internal mammary artery (LIMA) after coronary artery bypass grafting may lead to recurrent angina.

**Case Summary:**

A 64-year-old man presented with recurrent angina 2 years after coronary artery bypass grafting. Imaging showed perfusion defects in the territory of the left anterior descending artery (LAD). Coronary angiography revealed a large patent LIMA side branch. A percutaneous vascular plug successfully occluded the side branch, with symptom resolution.

**Discussion:**

There is conflicting evidence regarding the diagnosis and treatment of coronary steal syndrome from LIMA graft. Despite the lack of formal guidelines, reversible anterior wall defects on stress testing may help identify patients with clinically significant symptoms.

**Take-Home Messages:**

The side branches of the LIMA when unligated represent a relatively common anatomical variation that could have clinical implications. Large, unligated side branches when accompanied by anterior wall ischemia may indicate a diagnosis of LIMA-LAD steal amenable to percutaneous intervention.

## Graphical Abstract

**Figure:**
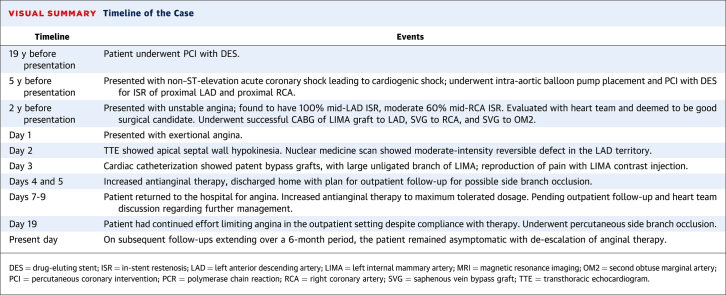

## History of Presentation

A 64-year-old man with established coronary artery disease (CAD) treated previously with drug-eluting stent and coronary artery bypass grafting (CABG) presented with angina and dyspnea. His vital signs were normotensive, with a regular heart rate; his cardiac examination was benign, without murmur or signs of heart failure.

**Take-Home Messages**

• In a post-CABG patient with otherwise patent bypass anatomy, consider LIMA steal syndrome given the presence of an unligated side branch.
• Not all cases are amenable to successful symptom-resolving intervention. Patient history along with objective multimodal diagnostics to look for ischemia can aid in the identification of this phenomenon.

## Past Medical History

**Figure 1:**
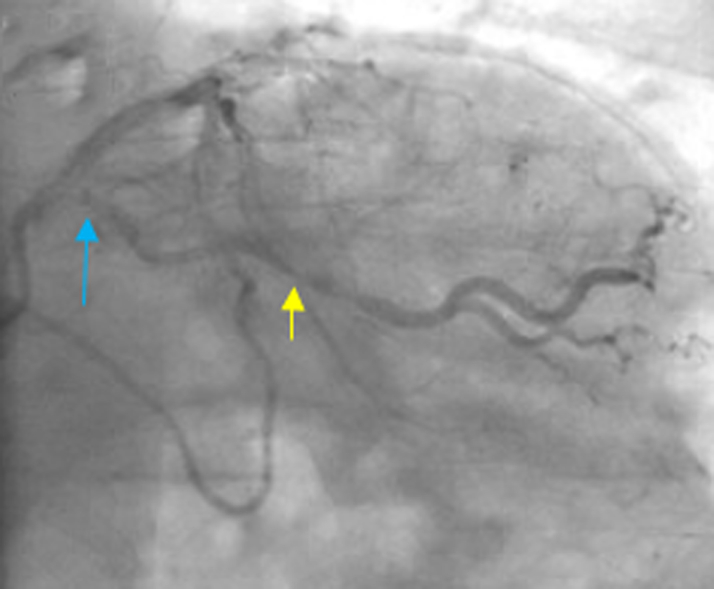
Coronary Angiography Before CABG Revascularization The image shows coronary artery disease in the left coronary system. There is a 60% ostial left circumflex lesion (blue arrow) and a 60% proximal OM2 disease (yellow arrow). CABG = coronary artery bypass grafting; OM2 = second obtuse marginal artery.

**Figure 2:**
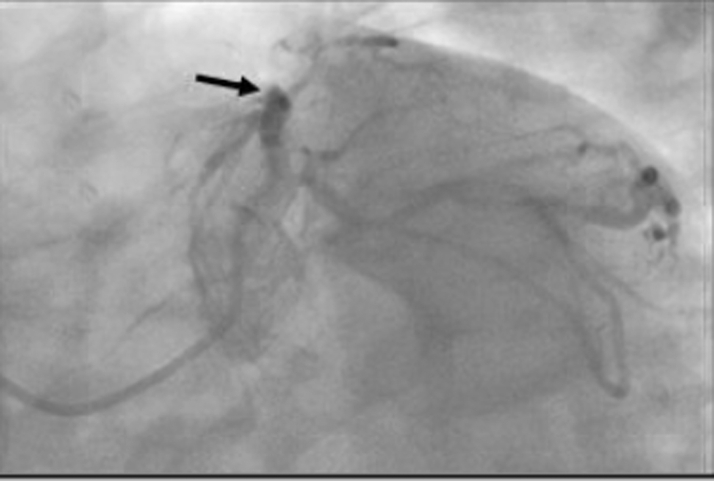
Coronary Angiography Before CABG Revascularization The image shows the coronary anatomy at the site of a previous percutaneous coronary intervention. There is 100% in-stent restenosis of the proximal LAD stent (arrow). CABG = coronary artery bypass grafting; LAD = left anterior descending artery.

**Figure 3:**
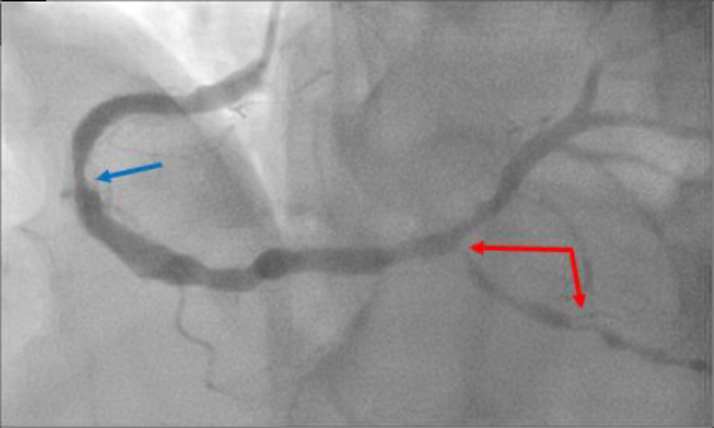
Coronary Angiography Before CABG Revascularization The image shows coronary artery disease in the right coronary system. There is 60% in-stent restenosis in the RCA (blue arrow) and 90% ostial and mid PDA lesion (red arrows). CABG = coronary artery bypass grafting; PDA = posterior descending artery; RCA = right coronary artery.

He had established CAD that had been treated with multiple prior percutaneous coronary interventions, with 7 drug-eluting stents placed over the span of 20 years and subsequent CABG revascularization for repeated events of severe in-stent restenosis and stent occlusion (Figure 1, Figure 2, Figure 3). Given multivessel disease with extensive prior percutaneous coronary interventions, a heart team approach was utilized, and the patient underwent CABG using left internal mammary artery (LIMA) graft to left anterior descending artery (LAD) as well as saphenous vein bypass grafts (SVGs) to the second obtuse marginal artery (OM2) and the distal right coronary artery (RCA).

## Differential Diagnosis

In this patient with a previous history of CABG revascularization, differential diagnosis included graft failure, in-stent restenosis, progression of CAD in the native vessels, coronary steal syndrome, and/or arrythmia leading to classic exertional symptoms. Other causes to consider for patients with chest pain and dyspnea included pericarditis, pulmonary embolism, and pericardial and pleural effusion.

## Investigations

**Figure 4:**
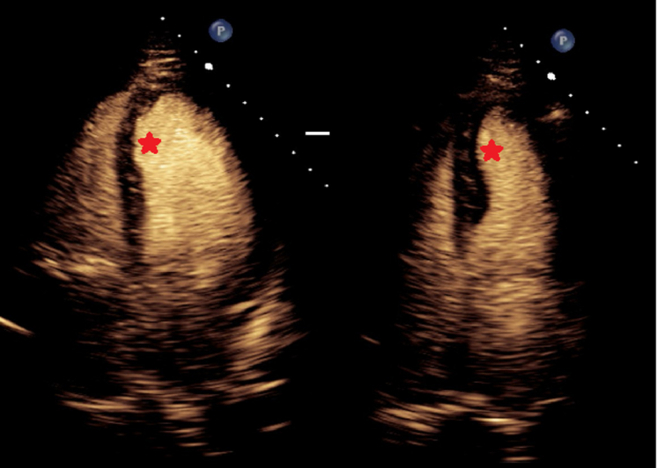
Transthoracic Echocardiogram With Contrast to Assess for Wall Motion Abnormality (Left) End-systole and (Right) end-diastole images with a hypokinetic segment of the apical septal wall and apex (red stars).

**Figure 5:**
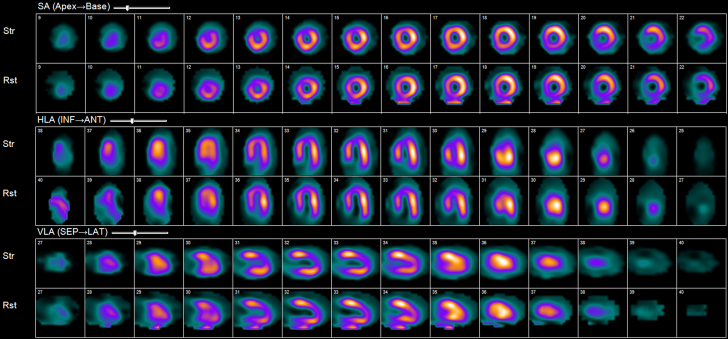
Nuclear Medicine Scans to Assess for Stress-Induced Ischemia There is a severe, partially reversible defect involving the mid-apical septal wall and apex, which is consistent with the vascular territory of the left anterior descending artery.

An electrocardiogram showed no ischemic changes and no changes from prior results. Transthoracic echocardiography (TTE) showed preserved ejection fraction with akinesis of the apical septal wall (Figure 4). A nuclear medicine cardiac stress test was then performed, which showed a severe, medium partially reversible defect in the mid, distal, and apical anterior walls in the distribution of the LAD territory (Figure 5).

**Figure 6:**
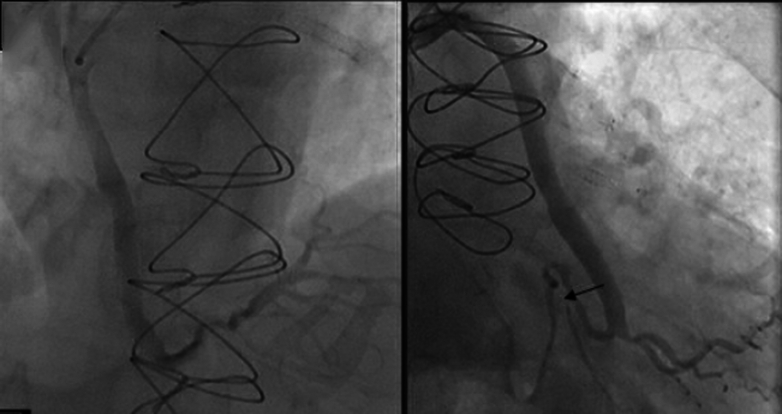
Cardiac Catheterization After Noninvasive Work-Up Coronary angiography was performed to establish the right coronary system native and bypass graft anatomy. (Left) Patent SVG to the RCA and diffuse PDA disease. (Right) Patent SVG to the OM2 with midsegment OM2 lesion proximal to the bypass anastomosis (arrow). OM2 = second obtuse marginal artery; PDA = posterior descending artery; RCA = right coronary artery; SVG = saphenous vein bypass graft.

**Figure 7:**
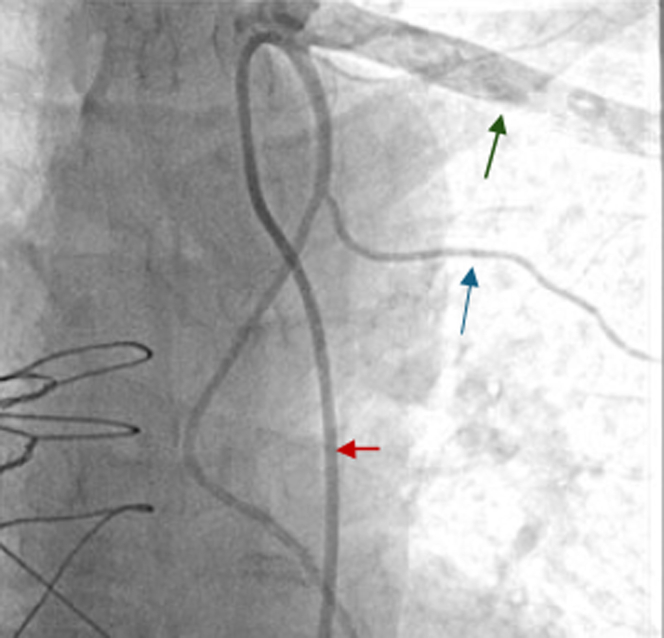
Cardiac Catheterization After Noninvasive Work-Up Coronary angiography was performed to establish the left coronary system native and bypass graft anatomy. A 6-F selective LIMA catheter (red arrow) engaging a patent LIMA branching from left subclavian artery (green arrow) with approximately 3-mm side branch (blue arrow). LIMA = left internal mammary artery.

Given the patient’s symptoms, along with findings of ischemia on stress nuclear medicine imaging and regional wall motion abnormality on TTE, we performed cardiac catheterization to define present coronary and bypass graft anatomy. Cardiac catheterization revealed unchanged native coronary anatomy. The SVGs to the OM2 and RCA were patent; the OM2 was noted to have a severe midsegment lesion. Diffuse disease was noted in the posterior descending artery in the right coronary system (Figure 6). The LIMA-to-LAD graft was still patent, with diffuse moderate disease distal to the anastomosis. LIMA angiography showed a large side branch of the LIMA that remained patent (Figure 7).

During the case, the patient developed transient chest pain with LIMA contrast injection. Given no obvious culprit lesion, he was treated with beta-blocker, long-acting nitrate, and ranolazine 1,000 mg twice daily. The patient was then discharged for outpatient follow-up, however he returned to the hospital for angina despite compliance with medical management.

## Management

Given catheterization findings of reproducible chest pain during angiography, a large, unligated side branch with known abnormal stress test in LAD distribution, and persistent angina, the decision was made to proceed with side-branch occlusion using a vascular plug.

**Figure 8:**
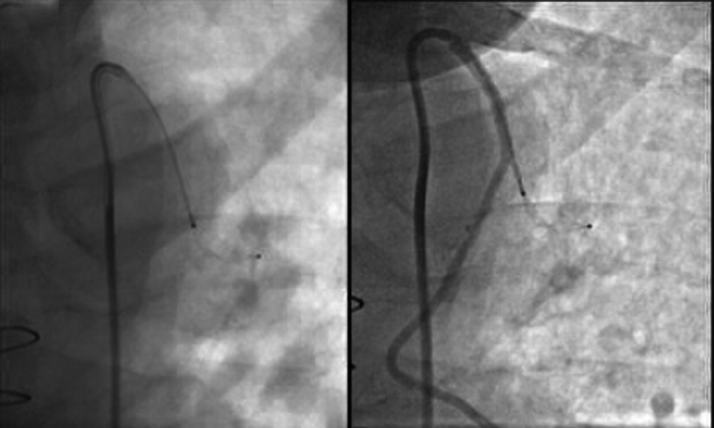
Percutaneous Intervention for Side Branch Occlusion (Left) A guide wire in a LIMA side branch. (Right) A 5-mm vascular plug that was placed over the wire can be seen occluding the proximal portion of the side branch with a patent LIMA. LIMA = left internal mammary artery.

For intervention, a 6-F × 90 cm Launcher guide catheter (Medtronic) was used to engage the LIMA. The side branch was felt to be 3 mm in diameter when compared with the 6-F guide catheter (Figure 7). Next, a 0.014 inch × 300 cm BMW Elite wire (Abbott) was placed in the proximal side branch (Figure 8). A 4-F × 120 cm GlideCath straight guiding catheter (Terumo) was placed in the proximal portion of the LIMA side branch. Given the estimated size of the side branch was approximately 3 mm, a 5-mm vascular plug was then loaded onto the 4-F GlideCath catheter and deployed 6 to 7 mm beyond the origin of the vessel (Figure 8). Complete occlusion was noted within 3 minutes, without any compromise to the LIMA graft. There was improved distal graft and LAD flow.

## Outcome and Follow-Up

The patient was evaluated over a 6-month period, with resolution of angina on decreasing antianginal therapy.

## Discussion

The LIMA conduit is preferred for LAD bypass, given superior long-term survival, graft patency, and decreased hospitalizations.^1^ Anatomically, the LIMA has several side branches to supply the chest wall, sternum, and surrounding structures. Ligation of these side branches is normally performed during bypass to prevent coronary steal from the LAD leading to cardiac ischemia. It should be noted that leaving unligated side branches of the LIMA during surgical revascularization is a technical error. It is important to be vigilant in identifying and ligating visible branches during conduit preparation. When possible, surgeons should avoid leaving unligated side branches to decrease the incidence of potentially avoidable complications in the form of coronary steal syndrome. Despite this, it is estimated that patent large side branches are present in up to 30% of CABG patients.^2^

There are numerous case series and case reports documenting successful intervention of LIMA side branches in resolving angina, however there remains significant controversy regarding the phenomenon's contribution to angina, given contradicting hemodynamic and angiographic findings. Luise et al^3^ evaluated intra-LIMA Doppler flow with and without large unligated side branches and found no differences in flow velocity before and after adenosine injection in intra-LIMA average peak resting velocities. Guzon et al^4^ published similar findings in 3 patients who underwent temporary balloon occlusion of side branch without differences in coronary flow reserve. A criticism of these studies, however, is that the patient populations were not controlled for the number or size of side branches.^5^ In contrast to these studies,^3^^,^^4^ evidence to support a hemodynamic and structural relationship between presence of a large unligated LIMA side branch and LAD coronary steal has been documented. Singh and Sosa^6^ showed a correlation between mean distal LIMA angiographic diameter and presence of large side branch.

Of interest, as was the case in our patient, is the presence of LAD territory ischemia. Similar cases exist; Abdo et al^7^ reported a case after a positive stress test in LAD territory in a CABG patient with a large LIMA side branch. Sawaya et al,^8^ again in contrast to the studies by Luise et al^3^ and Guzon et al,^4^ demonstrated coronary flow reserve improvement in response to adenosine hyperemic challenge with transient balloon occlusion in a patient with reversible LAD territory defect. A larger cohort of 407 patients after CABG was studied by Beton et al,^9^ who found that patients with documented anterior wall ischemia tended to have longer and wider side branches than those without anterior wall ischemia.Finally, a literature review by Mangels et al^5^ of 44 cases ranging from 1990 to 2018 found that 21 cases had documentation of anteriorr wall ischemia based on stress testing.

Although no formal guidelines exist for this rare entity, the presence of anterior defect on stress imaging in LAD territory appears to be a good objective measure for the diagnosis of LIMA-LAD steal syndrome in patients with angina post-CABG with identified large-caliber side branches.

## Conclusions

LIMA-LAD steal is a rare entity that continues to be controversial in physiologic and hemodynamic diagnosis. LIMA angiography paired with stress test has been documented in identifying this phenomenon in patients after CABG, and it may be a predictor of likelihood of successful intervention and outcome. Vascular plug occlusion is a possible minimally invasive treatment option for these patients.

**Visual Summary:** Timeline of the Case

Timeline	Events
19 y before presentation	Patient underwent PCI with DES.
5 y before presentation	Presented with non–ST-elevation acute coronary shock leading to cardiogenic shock; underwent intra-aortic balloon pump placement and PCI with DES for ISR of proximal LAD and proximal RCA.
2 y before presentation	Presented with unstable angina; found to have 100% mid-LAD ISR, moderate 60% mid-RCA ISR. Evaluated with heart team and deemed to be good surgical candidate. Underwent successful CABG of LIMA graft to LAD, SVG to RCA, and SVG to OM2.
Day 1	Presented with exertional angina.
Day 2	TTE showed apical septal wall hypokinesia. Nuclear medicine scan showed moderate-intensity reversible defect in the LAD territory.
Day 3	Cardiac catheterization showed patent bypass grafts, with large unligated branch of LIMA; reproduction of pain with LIMA contrast injection.
Days 4 and 5	Increased antianginal therapy, discharged home with plan for outpatient follow-up for possible side branch occlusion.
Days 7-9	Patient returned to the hospital for angina. Increased antianginal therapy to maximum tolerated dosage. Pending outpatient follow-up and heart team discussion regarding further management.
Day 19	Patient had continued effort limiting angina in the outpatient setting despite compliance with therapy. Underwent percutaneous side branch occlusion.
Present day	On subsequent follow-ups extending over a 6-month period, the patient remained asymptomatic with de-escalation of anginal therapy.

DES = drug-eluting stent; ISR = in-stent restenosis; LAD = left anterior descending artery; LIMA = left internal mammary artery; MRI = magnetic resonance imaging; OM2 = second obtuse marginal artery; PCI = percutaneous coronary intervention; PCR = polymerase chain reaction; RCA = right coronary artery; SVG = saphenous vein bypass graft; TTE = transthoracic echocardiogram.

**Equipment List:** Equipment for LIMA Side Branch Occlusion

• 5-F × 10 cm Micro Introducer Kit • 6-F × 12 cm Introducer sheath • 6-F × 90 cm IMA Launcher guiding catheter • 300 cm BMW Elite wire • 4-F × 120 cm hydrophilic straight guiding catheter • Vascular plug

## Funding Support and Author Disclosures

The authors have reported that they have no relationships relevant to the contents of this paper to disclose.
